# miR-29a-deficiency does not modify the course of murine pancreatic acinar carcinoma

**DOI:** 10.18632/oncotarget.15850

**Published:** 2017-03-02

**Authors:** James Dooley, Vasiliki Lagou, Josselyn E. Garcia-Perez, Uwe Himmelreich, Adrian Liston

**Affiliations:** ^1^ VIB Center for Brain and Disease Research, Leuven, Belgium; ^2^ KU Leuven-University of Leuven, Department of Microbiology and Immunology, Leuven, Belgium; ^3^ KU Leuven-University of Leuven, Department of Imaging and Pathology, Molecular Small Animal Imaging Center (MOSAIC), Leuven, Belgium

**Keywords:** pancreatic cancer, microRNA, miR-29, in vivo

## Abstract

The development of cancers involves the complex dysregulation of multiple cellular processes. With key functions in simultaneous regulation of multiple pathways, microRNA (miR) are thought to have important roles in the oncogenic formation process. miR-29a is among the most abundantly expressed miR in the pancreas. Together with altered expression in pancreatic cancer cell lines and biopsies, and known oncogenic functions in leukemia, this expression data has identified miR-29a as a key candidate for miR involvement in pancreatic cancer biology. Here we used miR-29a-deficient mice and the TAg model of pancreatic acinar carcinoma to functionally test the role of miR-29a *in vivo*. We found no impact of miR-29a loss on the development or growth of pancreatic tumours, nor on the survival of tumour-bearing mice. These results suggest that, despite differential expression, miR-29a is oncogenically neutral in the pancreatic acinar carcinoma context. If these results are extended to other models of pancreatic cancer, they would reduce the attractiveness of miR-29a as a potential therapeutic target in pancreatic cancer.

## INTRODUCTION

Pancreatic cancer is a relatively rare form of cancer, with a lifetime risk of developing pancreatic cancer of 1 in 76. However, due to the high fatality rate, pancreatic cancer is the fourth highest cancer in the absolute number of fatalities [1]. The main reason for high mortality is late detection, with only 15–20% of cases being diagnosed at a point when they remain resectable, leading to a median survival of less than six months and a five year survival rate of under 5% [2]. Late detection makes patient study complex, increasing the reliance on murine pancreatic cancer models, such as the well-characterised Ela1-TAg transgenic strain. These mice, developed on the C57BL/6 background, express the SV40 T antigen (TAg) under the control of the rat elastase 1 (*Ela1*) promoter, resulting in expression in pancreatic acinar cells. Initial characterization of the C57BL/6 transgenic strain demonstrated pancreatic dysplasia in the embryonic pancreas, with cancer progression after birth to primary pancreatic acinar cell tumor formation as early as 10 weeks of age, with mice moribund around 20–30 weeks of age [3, 4]. While the Ela1-TAg mice develop acinar carcinoma, and not the more common ductal adenocarcinoma, the properties of tumour development (synchronized development and growth) make it amenable to *in vivo* functional testing.

Recent research has highlighted the importance of microRNA (miR), small non-coding RNA capable of complex regulation, in the development of multiple different cancer types, including pancreatic cancer [5]. Pancreatic cancers develop with altered miR expression profiles compared to normal pancreatic tissue [6]. These miR changes may be non-causative, or they may aid the cancer growth by the upregulation of oncogenes or downregulation of tumour suppressors [7]. Interfering with miR biology has been proposed as a future therapeutic for pancreatic cancer, but this process first requires formal testing of which miR are oncomirs and anti-oncomirs [8], and which are oncogenically neutral.

miR-29a is an important miR of the miR-29 family, with important physiological functions in pancreatic biology [9]. In pancreatic cancer, miR-29a has been demonstrated to be down-regulated in pancreatic cancer cell-lines, and over-expression of miR-29a decreases proliferation, leading miR-29a to be labelled a tumour-suppressor-miR [10]. However, several studies suggest that the *in vivo* expression does not correlate with the *in vitro* results, as miR-29 is upregulated in pancreatic cancer surgical specimens [11, 12], indicating it as a potential oncomir. In other contexts, miR-29a has been demonstrated to be a *bona fide* oncomir. In leukemia miR-29a is upregulated in indolent human B cell chronic lymphocytic leukemia and acute myeloid leukemia, and spontaneous leukemia forms in mice which over-express miR-29a in B cells or myeloid cells [13–15].

With conflicting expression data on miR-29a in pancreatic cancer, and a demonstrated oncogenic function in other cancer types, it is important to direct test the function of miR-29a in *in vivo* murine models. Here we used miR-29a knockout mice and the TAg transgenic model of pancreatic acinar carcinoma to investigate the functional role of miR-29a in a mouse model of pancreatic cancer, using the acinar subtype. We found no functional role for miR-29a in the onset or growth of pancreatic acinar carcinoma, or in the death rate of tumour-bearing mice, indicating that miR-29a is oncogenically neutral.

## RESULTS

**Figure 1:**
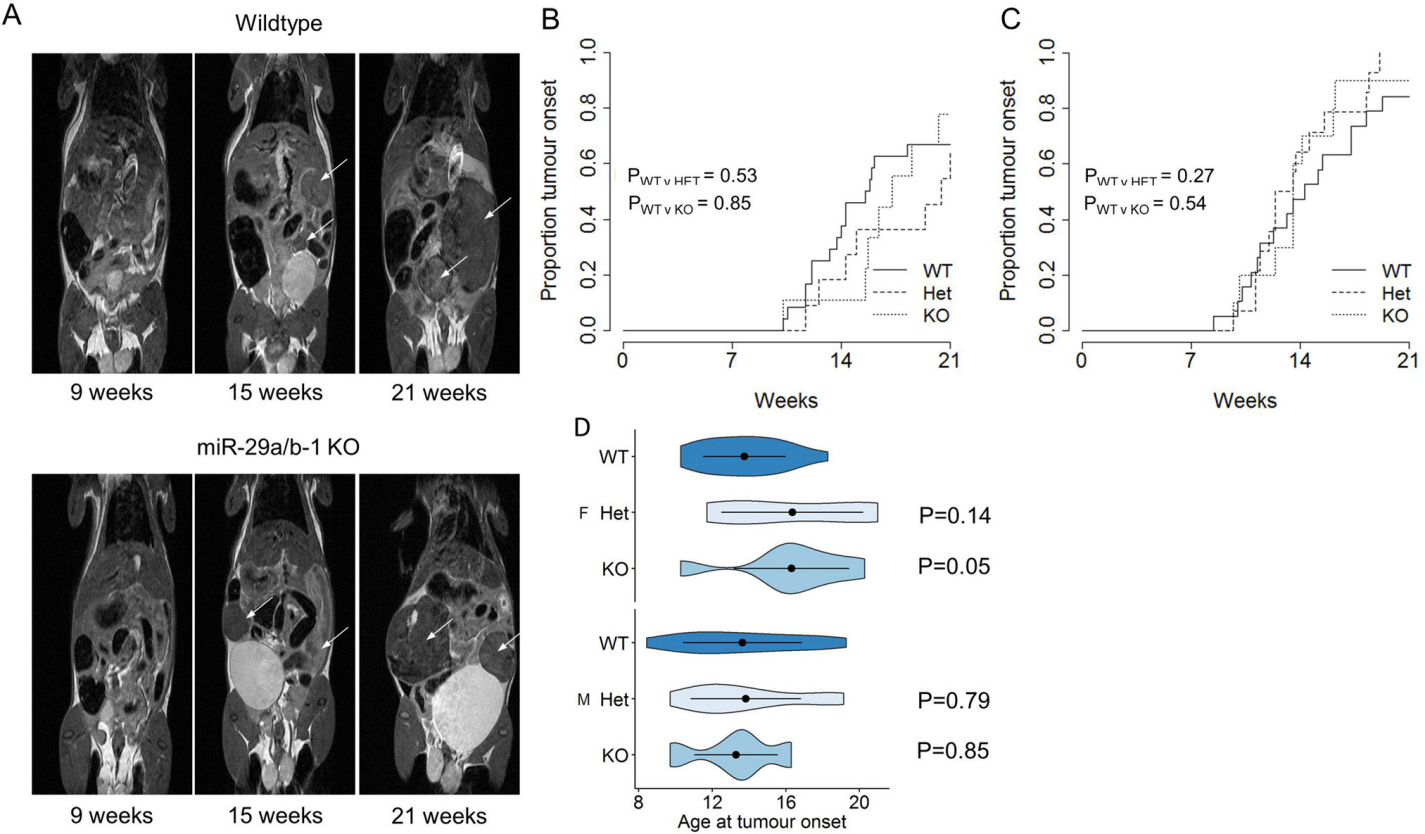
No effect of miR-29a on tumour onset in a pancreatic cancer model Ela1-TAg^+^ mice, on the wildtype, *miR-29a* heterozygous and miR-29a knockout backgrounds, were monitored for pancreatic cancer detection by MRI every two weeks. (**A**) Representative MRI scans for wildtype (top) and miR-29a knockout (bottom) mice, at 9 weeks, 15 weeks and 21 weeks. Arrows indicate detected tumours. (**B**) Cumulative incidence of pancreatic cancer as a function of age at tumour onset in wild-type, heterozygous and miR-29a-decifient mice, for female and (*n* = 24, 11, 9) (**C**) male (*n* = 21, 14, 10). The *P* values were calculated using the log-rank test. (**D**) Violin plots showing the mean, standard deviation and kernel probability density of the age at tumour onset under each condition in female (upper panel) and male (lower panel) mice. The *P* values were calculated using two-sided Mann-Whitney *U* test.

In order to directly test the *in vivo* function of miR-29a in pancreatic acinar carcinoma, we intercrossed the miR-29a-deficient mouse strain (deficient in both miR-29a and miR-29b-1) [16] with the spontaneous pancreatic acinar carcinoma Ela1-TAg transgenic strain described above [3]. TAg^+^ mice, wildtype, heterozygous or knockout for miR-29a, were monitored for pancreatic acinar carcinoma development through MRI assessment every two weeks, from the age of 7 weeks. MRI assessment allowed the detection of tumours (Figure 1A). For both female (Figure 1B) and male (Figure 1C) mice, no significant difference was observed in the cumulative incidence of pancreatic acinar carcinoma. When assessing the age of first tumour detection, no significant effect of miR-29a genotype was observed for male mice (Figure 1D), with only a minor impact of delayed tumour onset observed for female mice (Figure 1D).

**Figure 2:**
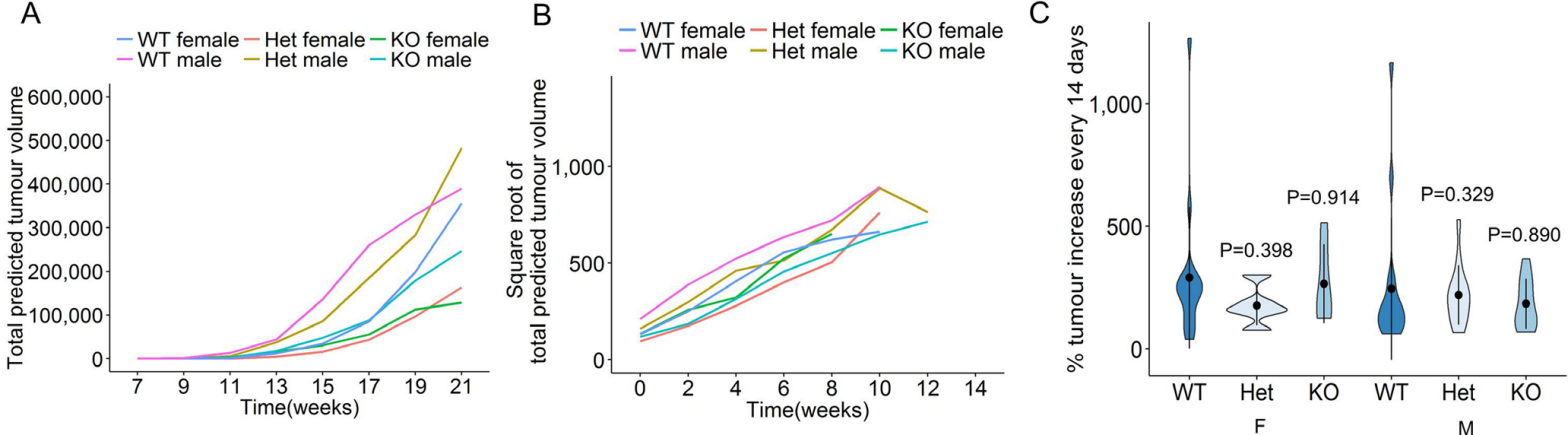
No effect of miR-29a on tumour growth in a pancreatic cancer model Ela1-TAg^+^ mice, on the wildtype, *miR-29a* heterozygous and miR-29a knockout backgrounds, were monitored for pancreatic cancer growth by MRI every two weeks. (**A**) Total tumour volume was estimated at each time-point for wildtype, heterozygous and knockout female mice, and wildtype, heterozygous and knockout male mice (*n* = 24, 11, 9, 21, 14, 10). Each line indicates average tumour size across the group. (**B**) Individual square root transformed total predicted tumour volume curves for wildtype, heterozygous and knockout female and male mice (*n* = 24, 11, 9, 21, 14, 10). Time 0 corresponds to the first detected tumour time-point and each line indicates tumour size in a single mouse. (**C**) Violin plots showing the mean, standard deviation and kernel probability density of the % tumour increase every two weeks under each condition in female and male mice (*n* = 24, 11, 9, 21, 14, 10). The *P* values were calculated using two-sided Mann-Whitney *U* test.

**Figure 3:**
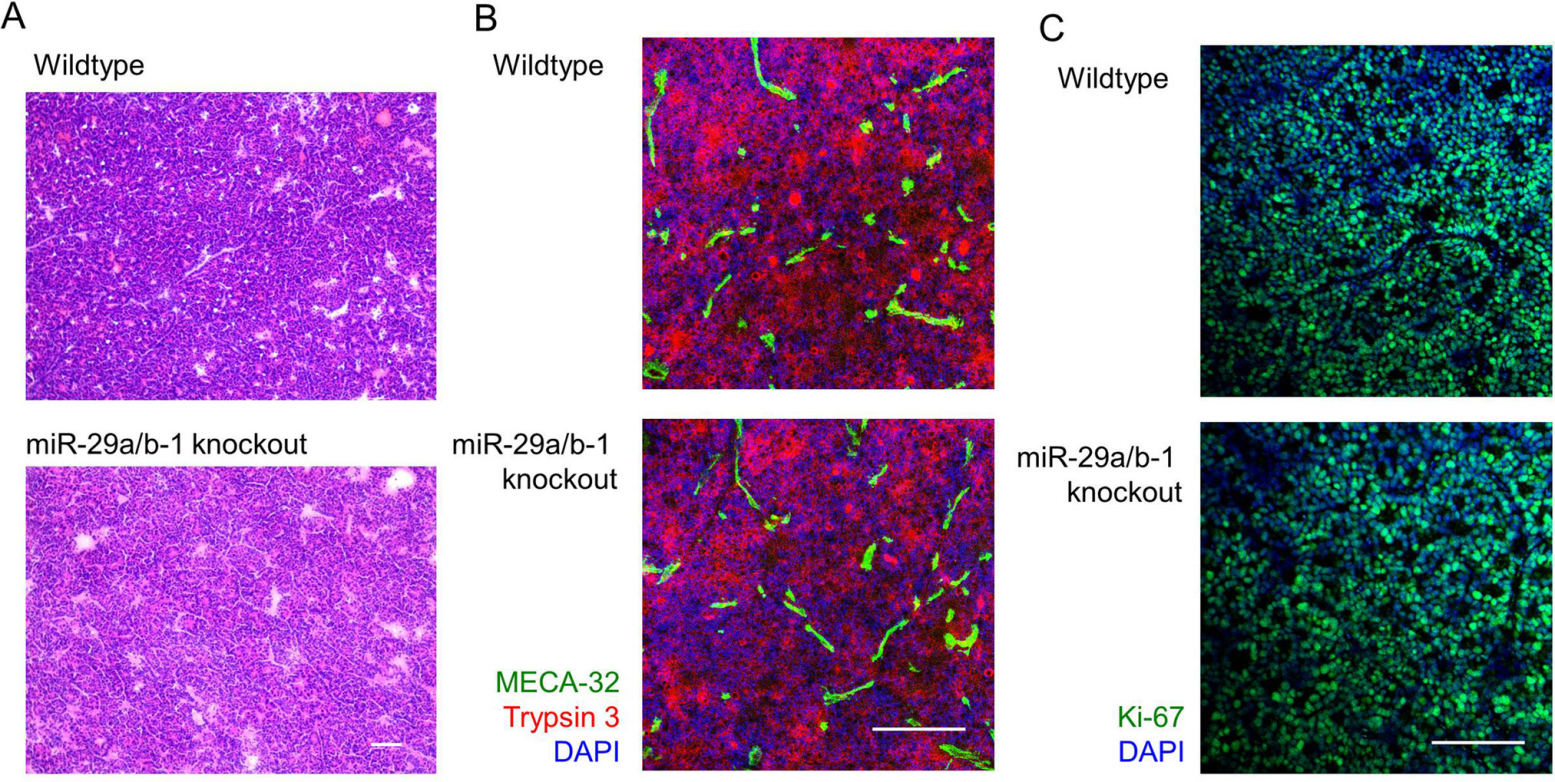
miR-29a-deficient tumours present with normal histopathological growth Ela1-TAg^+^ mice, on the wildtype and miR-29a knockout backgrounds were followed to 21 weeks of age, at which point tumours assessed by fresh frozen histology. (**A**) H&E histological assessment. Scale = 100 μm. (**B**) Trypsin 3 (acinar cell carcinoma marker), MECA-32 (vascularisation marker) and DAPI staining by immunofluorescence. Scale = 50 μm. (**C**) Ki67 (proliferation marker) and DAPI staining by immunofluorescence. Scale = 50 μm. Representative images of *n* = 3/group displayed.

To determine the impact of miR-29a on pancreatic acinar carcinoma growth post-development, *miR-29a* wildtype, heterozygous and knockout mice were longitudinally monitored from first cancer detection to death, excessive morbidity or 21 weeks of age. MRI assessment allowed longitudinal tumour growth tracking. Within each individual mouse the total number of tumours and cross-sectional maximal size was measured, allowing the calculation of total tumour volume. Despite the variation in first tumour detection, post-detection each tumour grew in a classical exponential growth fashion, regardless of sex or miR-29a genotype (Supplementary Figure 1, Figure 2A). A linear mixed-effect model found no significant differences in tumour curves. In order to directly compare the growth rates of tumours within each mouse, we square root transformed total tumour volume and plotted tumour growth from time of first detection (Supplementary Figure 2, Figure 2B). Direct comparison of tumour growth rates was performed as the percentage of tumour volume increase between MRI measurement (every 14 days, averaged per mouse over the entire course of tumour measurement). For both male and female mice, no change in the tumour growth rate was observed in miR-29a heterozygous or miR-29a knockout mice (Figure 2C). As an independent approach, we assessed tumours from wildtype and knockout mice by histology and immunofluorescence, observing no consistent differences (Figure 3A, 3B). Proliferation assessment through Ki67 demonstrated similar rates of proliferation across the genotypes (Figure 3C), providing both biochemical and MRI support an oncogenically neutral role of this biomarker miR.

**Figure 4:**
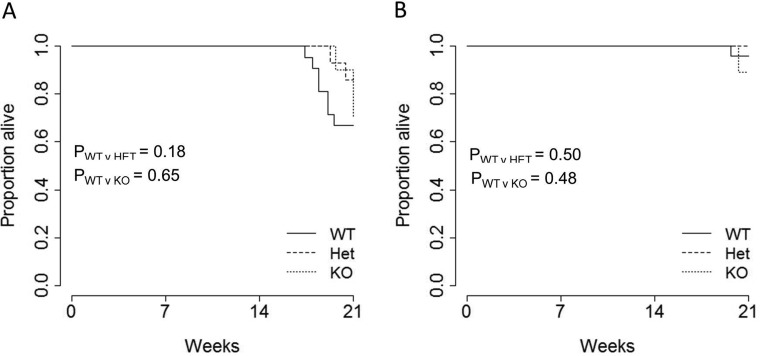
No effect of miR-29a on survival in a pancreatic cancer model Ela1-TAg^+^ mice, on the wildtype, *miR-29a* heterozygous and miR-29a knockout backgrounds, were monitored for survival until the age of 21 weeks. Kaplan-Meier plots showing the overall pancreatic cancer survival in wild-type, heterozygous and miR-29a-deficient mice for both (**A**) male (*n* = 21, 14, 10) and (**B**) female (*n* = 24, 11, 9) mice. *P* values were calculated using the log-rank test.

Finally, to quantify the net impact of miR-29 genotype on pancreatic cancer-induced mortality, we measured overall pancreatic cancer survival. Survival was only measured out to 21 weeks, after which miR-29a-deficient mice exhibit enhanced mortality independent of pancreatic acinar carcinoma [9]. During this measurement period, the TAg transgene on the miR-29a wildtype background induced excessive mortality in male (Figure 4A), but not female (Figure 4B) mice. Loss of miR-29a, in either the heterozygous or homozygous state, did not significantly alter this survival.

## DISCUSSION

miR represent attractive therapeutic targets in pancreatic cancer [17]. Unlike protein-based biologics, RNA-based biologics are cheap and straightforward to manufacture. miR can be targeted to the relevant cells through either mechanical or viral-based delivery systems, and are able to simultaneously target multiple biologically relevant pathways. The ability to regulate hundreds of genes potentially makes miR treatment more difficult for cancers to evolve treatment resistance to. Both onco-miR and tumour-suppressor-miR represent potential targets, with the use of “miR sponges” representing a suitable mechanism to antagonise onco-miR [18], and direct miR supplementation being suitable to replace lost tumour-suppressor miR [19]. As with other oncogenes and tumour-suppressor genes, a key requirement to the therapeutic targeting is to determine whether the identified expression change has functional consequences on the cell – miR expression is highly sensitive to contextual change, and thus many identified expression changes may be oncogenically neutral rather than participating in the oncognenic event they are associated with.

Prior to this study, miR-29a was an attractive potential therapeutic target in pancreatic cancer. miR-29a |is known to maintain self-renewal capacity in haematopoietic stem cells [20], a property which leads to oncogenic potential when over-expressed in the B cell progeny [13, 14]. Targets of miR-29a include key pro-survival genes (Bcl2 and Mcl1) [21], and elevated miR-29c expression (with the same regulation-inducing seed sequence as miR-29a) is associated with resistance to treatment [22]. The *bona fide* oncomir activity of miR-29a in leukemia indicates that the observed upregulation in pancreatic cancer surgical specimens [11, 12] may reflect a similar oncomir property in pancreatic cancer. However *in vitro* experiments have suggested the converse, that miR-29a is a tumour-suppressor-miR in pancreatic cancer. miR-29a is downregulated in pancreatic cancer cell-lines, a change which elevates invasiveness and proliferation [10], in part by increasing MUC1 expression [23]. Again, parallels in other cell types support a potential tumour-suppressor-miR role for miR-29a. In acute myeloid leukemia, miR-29b-1 (part of the miR-29a cluster, and knocked out in the miR-29a-deficient mice) is downregulated, contributing to the loss of normal granulopoiesis [24], and likewise in thyroid cancer miR-29a appears to be a tumour-suppressor-miR, with loss removing AKT3 regulation [25]. Complementary tumour suppressor functions have been observed in squamous cell carcinoma [26, 27], among other cancer cell types. Despite the firm theoretical and *in vitro* basis for suggesting a functional role of miR-29a in pancreatic cancer, *in vivo* experimental validation was necessary before developing pre-clinical treatment strategies, especially as studies on the physiological role of pancreatic miR-29a expression have shown stark contradictions between *in vivo* and *in vitro* systems [9].

Our data firmly rule out a functional role for the *miR-29a* cluster (including miR-29a and miR-29b-1) in the development, growth and pathology of pancreatic acinar carcinoma in the murine Ela1-TAg model. There are distinct caveats to this model, most notably that acinar tumours occur only in the minority of pancreatic cancer patients. Furthermore, the genetic insult used to promote tumour formation in these mice is relatively crude, compared to more sophisticated models that have since become available that closely mimic the oncoformative events in patients [28]. It is possible that other pancreatic cancer models will end up demonstrating a functional role for miR-29a in tumour development or growth, or, indeed, that a species difference exists between mice and humans. Nonetheless, these results sharply reduce the attractiveness of miR-29a as a pancreatic cancer therapeutic target, without diminishing the potential diagnostic value of the observed altered expression which spurred the initiation of this study.

## MATERIALS AND METHODS

### Mice

miR-29a knockout mice [16] were intercrossed with mice bearing a transgene for the SV40 large T Antigen with an Elastase-1 mouse acinar cell promoter (Ela1-TAg), purchased from Jackson on the C57BL/6 background [3, 4]. Mice were bred under specific pathogen–free conditions and house under conventional conditions during magnetic resonance imaging (MRI), were fed using R/M-H ssniff chow and were used in accordance with the University of Leuven Animal Ethics Committee. Mouse-weight and blood glucose were monitored throughout the experimental process.

### Imaging

TAg^+^ mice were scanned every two weeks from 7 weeks of age. Mice were anesthetized using isoflurane (2% in 100% oxygen) during scan time. The temperature and respiration of anesthetized mice were monitored and maintained at 37°C and > 40 min-1 respectively. Images were acquired using a Bruker Biospin 9.4 Biospec Tesla small animal scanner (Bruker Biospin, Ettlingen, Germany) equipped with an actively shielded gradient set of 600mT/m using a respiration triggered spin echo sequence (RARE) with 50 continuous slices of 0.5 mm thickness in interlaced mode (TR = 6000 ms, TE = 15.9 ms, FOV = 4.0 × 6.0 cm, a matrix of 200 × 400, two dummy scans and two averages). For RF irradiation and detection a 7.2 cm quadrature resonator (Bruker Biospin, Ettlingen) was used.

### Histology

Histology fresh frozen sections were fixed in 4% PFA and stained followed by hematoxylin and eosin staining. For immunofluorescence, pancreatic tumours were fresh frozen in OCT, fixed in 4% PFA or acetone, and stained according to manufacturer's protocol. Sections were stained using the polyclonal Ki67 (Rabbit Anti-Ki67, Abcam ab15580), Trypsin 3/PRSS3 (Goat anti-Trypsin, R&D Systems AF3565) and MECA-32 (Rat anti-PLVAP, in-house hybridoma supernatant). For immunofluorescence the following detection antibody were used: Donkey anti-Rabbit 488 (Life Technologies), Donkey anti-Goat Alexa Fluor 546 (Life Technologies), Donkey anti-Rat 488 (Life Technologies) and DAPI (Life Technologies). Images were acquired using a Ziess LSM 510 meta confocal microscope.

### Statistical analysis

Tumour incidence was indicated when detectable tumour was visible on the MRI scan. Experimental endpoints were achieved and recorded at death, degeneration of mouse health below ethical threshold, aging past 21 weeks. MRI images were analysed using ImageJ software and the mean area was calculated at the maximum radius. The following calculation was used to predict tumour volume based on this value: 4/3*area*√(area/π). Data were collated and stored in Microsoft Excel. All calculations were made using R (www.r-project.org).

Tumour growth curves where analysed with a linear mixed-effect model, in which we included the cross level interaction between time and diet (i.e. the effect of time is allowed to vary between diet groups and across individuals). The formula of the model is as follows: tumour growth ∼ time + diet + time*diet+ (1+time|subject). This model provides a fixed-effect estimate for the interaction between change over time and diet that indicates whether the rate of change with respect to tumour growth is significantly different between the regular water and other diets. We considered the regular water diet as reference diet and week 7 as reference time. This linear mixed-effect models was fitted within each sex using the lmer function within lme4 (Linear Mixed-Effects Models using ‘Eigen’ and S4) package in R.

We computed estimates of a survival curve for censored data using the Kaplan-Meier estimator [29] that makes no parametric assumptions about the form of distribution (R package “npsurv”). Cumulative incidence curves were generated using the R package “survplot” with the fun=function(x) {1 − x} argument [30]. The comparison of cumulative incidence and survival distributions between two samples was performed using log-rank test implemented in the R “survdiff” package [31]. The logrank test is a non-parametric test and the most widely used method for comparing two or more survival curves [32]. Two group comparisons presented in the violin plots were made using two-sided Mann-Whitney *U* test.

## SUPPLEMENTARY MATERIALS FIGURES



## References

[1] Jemal A, Siegel R, Ward E, Hao Y, Xu J, Thun MJ (2009). Cancer statistics, 2009. CA Cancer J Clin.

[2] Cleary SP, Gryfe R, Guindi M, Greig P, Smith L, Mackenzie R, Strasberg S, Hanna S, Taylor B, Langer B, Gallinger S (2004). Prognostic factors in resected pancreatic adenocarcinoma: analysis of actual 5-year survivors. J Am Coll Surg.

[3] Tevethia MJ, Bonneau RH, Griffith JW, Mylin L (1997). A simian virus 40 large T-antigen segment containing amino acids 1 to 127 and expressed under the control of the rat elastase-1 promoter produces pancreatic acinar carcinomas in transgenic mice. J Virol.

[4] Ornitz DM, Hammer RE, Messing A, Palmiter RD, Brinster RL (1987). Pancreatic neoplasia induced by SV40 T-antigen expression in acinar cells of transgenic mice. Science.

[5] Yonemori K, Kurahara H, Maemura K, Natsugoe S (2016). MicroRNA in pancreatic cancer. J Hum Genet.

[6] Piepoli A, Tavano F, Copetti M, Mazza T, Palumbo O, Panza A, di Mola FF, Pazienza V, Mazzoccoli G, Biscaglia G, Gentile A, Mastrodonato N, Carella M (2012). Mirna expression profiles identify drivers in colorectal and pancreatic cancers. PLoS ONE.

[7] Jiao LR, Frampton AE, Jacob J, Pellegrino L, Krell J, Giamas G, Tsim N, Vlavianos P, Cohen P, Ahmad R, Keller A, Habib NA, Stebbing J (2012). MicroRNAs targeting oncogenes are down-regulated in pancreatic malignant transformation from benign tumors. PLoS ONE.

[8] Singh PK, Brand RE, Mehla K (2012). MicroRNAs in pancreatic cancer metabolism. Nat Rev Gastroenterol Hepatol.

[9] Dooley J, Garcia-Perez JE, Sreenivasan J, Schlenner SM, Vangoitsenhoven R, Papadopoulou AS, Tian L, Schonefeldt S, Serneels L, Deroose C, Staats KA, Van der Schueren B, De Strooper B (2016). The microRNA-29 Family Dictates the Balance Between Homeostatic and Pathological Glucose Handling in Diabetes and Obesity. Diabetes.

[10] Muniyappa MK, Dowling P, Henry M, Meleady P, Doolan P, Gammell P, Clynes M, Barron N (2009). MiRNA-29a regulates the expression of numerous proteins and reduces the invasiveness and proliferation of human carcinoma cell lines. Eur J Cancer.

[11] Roldo C, Missiaglia E, Hagan JP, Falconi M, Capelli P, Bersani S, Calin GA, Volinia S, Liu CG, Scarpa A, Croce CM (2006). MicroRNA expression abnormalities in pancreatic endocrine and acinar tumors are associated with distinctive pathologic features and clinical behavior. J Clin Oncol.

[12] Zhang Y, Li M, Wang H, Fisher WE, Lin PH, Yao Q, Chen C (2009). Profiling of 95 microRNAs in pancreatic cancer cell lines and surgical specimens by real-time PCR analysis. World J Surg.

[13] Santanam U, Zanesi N, Efanov A, Costinean S, Palamarchuk A, Hagan JP, Volinia S, Alder H, Rassenti L, Kipps T, Croce CM, Pekarsky Y (2010). Chronic lymphocytic leukemia modeled in mouse by targeted miR-29 expression. Proc Natl Acad Sci U S A.

[14] Han YC, Park CY, Bhagat G, Zhang J, Wang Y, Fan JB, Liu M, Zou Y, Weissman IL, Gu H (2010). microRNA-29a induces aberrant self-renewal capacity in hematopoietic progenitors, biased myeloid development, and acute myeloid leukemia. J Exp Med.

[15] Liston A, Papadopoulou AS, Danso-Abeam D, Dooley J (2012). MicroRNA-29 in the adaptive immune system: setting the threshold. Cell Mol Life Sci.

[16] Papadopoulou AS, Dooley J, Linterman MA, Pierson W, Ucar O, Kyewski B, Zuklys S, Hollander GA, Matthys P, Gray DH, De Strooper B, Liston A (2012). The thymic epithelial microRNA network elevates the threshold for infection-associated thymic involution via miR-29a mediated suppression of the IFN-alpha receptor. Nat Immunol.

[17] Taucher V, Mangge H, Haybaeck J (2016). Non-coding RNAs in pancreatic cancer: challenges and opportunities for clinical application. Cell Oncol (Dordr).

[18] Jung J, Yeom C, Choi YS, Kim S, Lee E, Park MJ, Kang SW, Kim SB, Chang S (2015). Simultaneous inhibition of multiple oncogenic miRNAs by a multi-potent microRNA sponge. Oncotarget.

[19] Kota J, Chivukula RR, O'Donnell KA, Wentzel EA, Montgomery CL, Hwang HW, Chang TC, Vivekanandan P, Torbenson M, Clark KR, Mendell JR, Mendell JT (2009). Therapeutic microRNA delivery suppresses tumorigenesis in a murine liver cancer model. Cell.

[20] Hu W, Dooley J, Chung SS, Chandramohan D, Cimmino L, Mukherjee S, Mason CE, de Strooper B, Liston A, Park CY (2015). miR-29a maintains mouse hematopoietic stem cell self-renewal by regulating Dnmt3a. Blood.

[21] Xu L, Xu Y, Jing Z, Wang X, Zha X, Zeng C, Chen S, Yang L, Luo G, Li B, Li Y (2014). Altered expression pattern of miR-29a, miR-29b and the target genes in myeloid leukemia. Exp Hematol Oncol.

[22] Butrym A, Rybka J, Baczynska D, Poreba R, Kuliczkowski K, Mazur G (2016). Clinical response to azacitidine therapy depends on microRNA-29c (miR-29c) expression in older acute myeloid leukemia (AML) patients. Oncotarget.

[23] Trehoux S, Lahdaoui F, Delpu Y, Renaud F, Leteurtre E, Torrisani J, Jonckheere N, Van Seuningen I (2015). Micro-RNAs miR-29a and miR-330-5p function as tumor suppressors by targeting the MUC1 mucin in pancreatic cancer cells. Biochim Biophys Acta.

[24] Eyholzer M, Schmid S, Wilkens L, Mueller BU, Pabst T (2010). The tumour-suppressive miR-29a/b1 cluster is regulated by CEBPA and blocked in human AML. Br J Cancer.

[25] Li R, Liu J, Li Q, Chen G, Yu X (2016). miR-29a suppresses growth and metastasis in papillary thyroid carcinoma by targeting AKT3. Tumour Biol.

[26] Yamamoto N, Kinoshita T, Nohata N, Yoshino H, Itesako T, Fujimura L, Mitsuhashi A, Usui H, Enokida H, Nakagawa M, Shozu M, Seki N (2013). Tumor-suppressive microRNA-29a inhibits cancer cell migration and invasion via targeting HSP47 in cervical squamous cell carcinoma. Int J Oncol.

[27] Kinoshita T, Nohata N, Hanazawa T, Kikkawa N, Yamamoto N, Yoshino H, Itesako T, Enokida H, Nakagawa M, Okamoto Y, Seki N (2013). Tumour-suppressive microRNA-29s inhibit cancer cell migration and invasion by targeting laminin-integrin signalling in head and neck squamous cell carcinoma. Br J Cancer.

[28] Westphalen CB, Olive KP (2012). Genetically engineered mouse models of pancreatic cancer. Cancer J.

[29] Kaplan EL, Meier P (1958). Nonparametric-Estimation from Incomplete Observations. Journal of the American Statistical Association.

[30] Teno JM, Harrell FE, Knaus W, Phillips RS, Wu AW, Connors A, Wenger NS, Wagner D, Galanos A, Desbiens NA, Lynn J (2000). Prediction of survival for older hospitalized patients: the HELP survival model. Hospitalized Elderly Longitudinal Project. J Am Geriatr Soc.

[31] Harrington DP, Fleming TR (1982). A Class of Rank Test Procedures for Censored Survival-Data. Biometrika.

[32] Peto R, Pike MC, Armitage P, Breslow NE, Cox DR, Howard SV, Mantel N, McPherson K, Peto J, Smith PG (1977). Design and analysis of randomized clinical trials requiring prolonged observation of each patient. II. analysis and examples. Br J Cancer.

