# Supplementary material for: miR-29a-deficiency does not modify the course of murine pancreatic acinar carcinoma
Source: Oncotarget. 2017 Mar 2;8(16):26911–7. doi: 10.18632/oncotarget.15850 (PMC5432306; doi:10.18632/oncotarget.15850)
Supplement: Supplementary file 1 [file oncotarget-08-26911-s001.pdf]

# miR-29a-deficiency does not modify the course of murine pancreatic acinar carcinoma

## Supplementary Materials

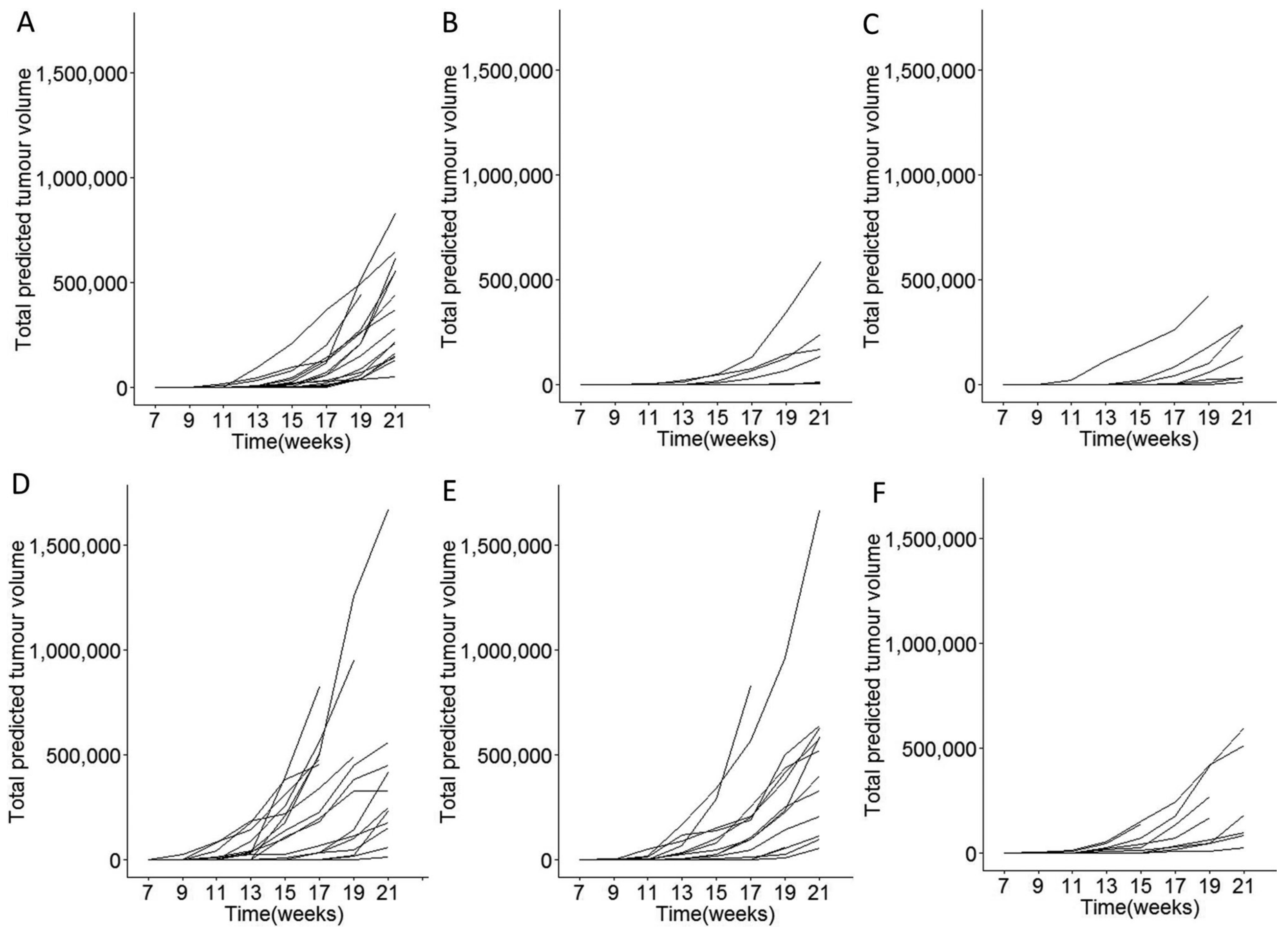


**Supplementary Figure 1: Longitudinal monitoring of tumour growth in miR-29a-deficient mice.** Ela1-TAg+ mice, on the wildtype, *miR-29a* heterozygous and miR-29a knockout backgrounds, were monitored for pancreatic cancer growth by MRI every two weeks. Total tumour volume was estimated at each time-point for (**A**) wildtype Ela1-TAg+ female mice, (**B**) *miR-29a* heterozygous Ela1-TAg+ female mice, (**C**) *miR-29a* knockout Ela1-TAg+ female mice, (**D**) wildtype Ela1-TAg+ male mice, (**E**) *miR-29a* heterozygous Ela1-TAg+ male mice, and (**F**) *miR-29a* knockout Ela1-TAg+ male mice ($n$ = 24, 11, 9, 21, 14, 10). Each line indicates tumour size in a single mouse.

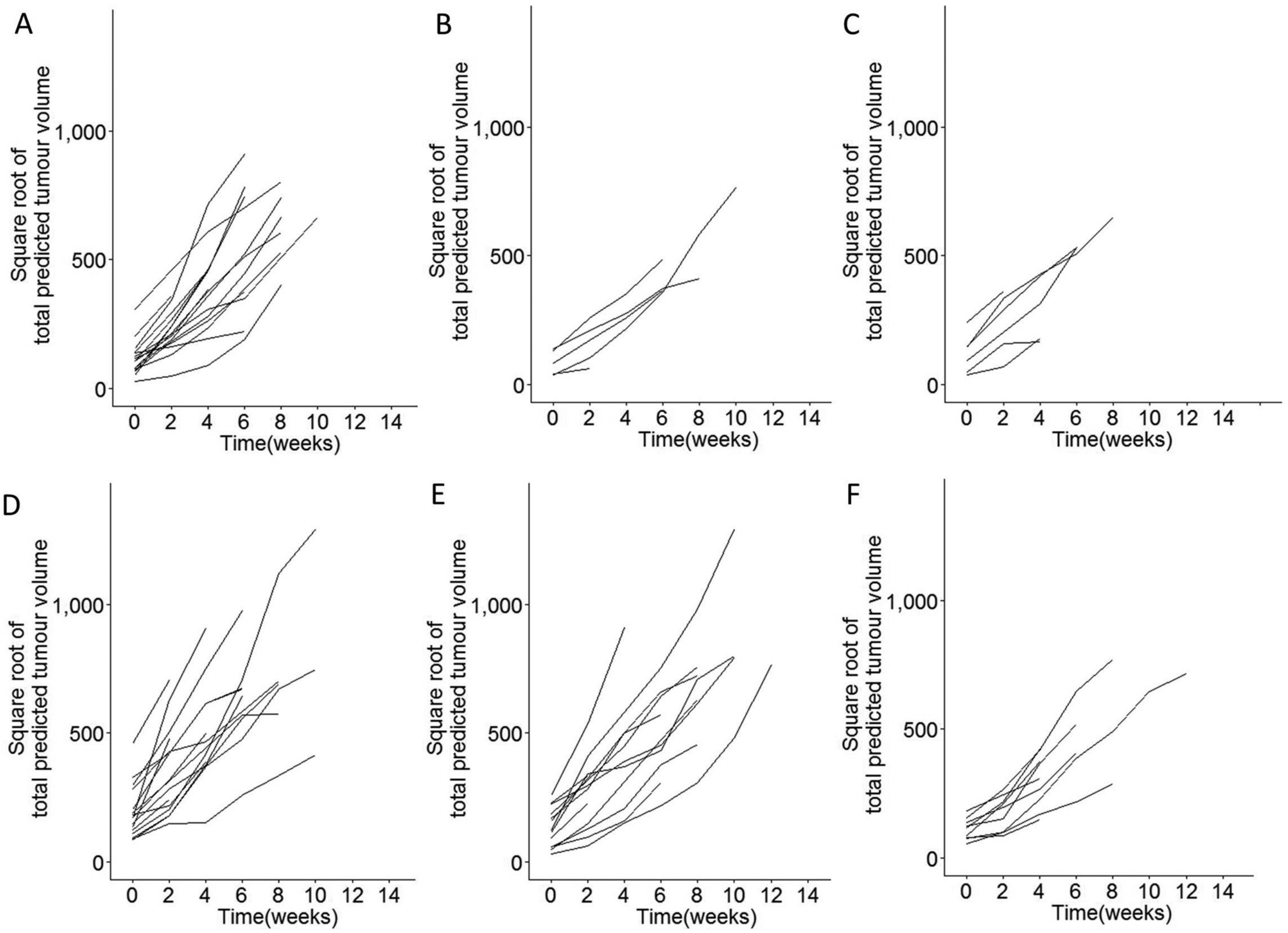


**Supplementary Figure 2: Calculation of tumour growth in a pancreatic cancer model.** Ela1-TAg$^{+}$ mice, on the wildtype, *miR-29a* heterozygous and miR-29a knockout backgrounds, were monitored for pancreatic cancer growth by MRI every two weeks. Individual square root transformed total predicted tumour volume curves for (**A**) wild-type female mice ($n$ = 24), (**B**) *miR-29a* heterozygous female mice ($n$ = 11), (**C**) *miR-29a*-deficient female mice ($n$ = 9), (**D**) wild-type male mice ($n$ = 21), (**E**) *miR-29a* heterozygous male mice ($n$ = 14) and (**F**) *miR-29a*-deficient male mice ($n$ = 10). Time 0 corresponds to the first detected tumour time-point and each line indicates tumour size in a single mouse.
